# CXCL9 and CXCL10 accelerate acute transplant rejection mediated by alloreactive memory T cells in a mouse retransplantation model

**DOI:** 10.3892/etm.2014.1714

**Published:** 2014-05-14

**Authors:** JIAWEI ZHUANG, ZHONGGUI SHAN, TENG MA, CHUN LI, SHUIWEI QIU, XIAOBIAO ZHOU, LIANFENG LIN, ZHONGQUAN QI

**Affiliations:** 1 Department of Cardiac Surgery, The First Affiliated Hospital, Xiamen University, Xiamen, Fujian 361003, P.R. China; 2 Organ Transplantation Institute, Medical College, Xiamen University, Xiamen, Fujian 361005, P.R. China

**Keywords:** C-X-C motif chemokine ligand 9, C-X-C motif chemokine ligand 10, retransplantation, memory T cells, heart transplantation

## Abstract

C-X-C motif chemokine ligand (CXCL) 9 and CXCL10 play key roles in the initiation and development of acute transplant rejection. Previously, higher levels of RANTES expression and secretion were demonstrated in retransplantation or T-cell memory-transfer models. In the present study, the effect of the chemokines, CXCL9 and CXCL10, were investigated in a mouse retransplantation model. BALB/c mice were used as donors, while C57BL/6 mice were used as recipients. In the experimental groups, a heterotopic heart transplantation was performed six weeks following skin grafting. In the control groups, a heterotopic heart transplantation was performed without skin grafting. Untreated mice served as blank controls. The mean graft survival time of the heterotopic heart transplantations was 7.7 days in the experimental group (n=6), as compared with 3.25 days in the control group (n=6; P<0.001). On day three following cardiac transplantation, histological evaluation of the grafts revealed a higher International Society for Heart & Lung Transplantation grade in the experimental group as compared with the control group. In addition, gene expression and serum concentrations of CXCL9, CXCL10, interferon-γ, and interleukin-2 were markedly higher in the experimental group when compared with the control group. Differences between the levels of CXCL9 and CXCL10 in the pre- and post-transplant mice indicated that the chemokines may serve as possible biomarkers to predict acute rejection. The results of the present study demonstrated that CXCL9 and CXCL10 play a critical role in transplantation and retransplantation. High levels of these cytokines during the pre-transplant period may lead to extensive acute rejection. Thus, the observations enhance the understanding of the mechanism underlying the increased expression and secretion of CXCL9 and CXCL10 by alloreactive memory T cells.

## Introduction

In adult humans, 40–50% of peripheral blood T cells have memory phenotypes (1), which can threaten graft survival (2,3). It is hypothesized that continuous exposure to bacterial and viral pathogens, blood transfusion or pregnancy may develop alloreactive memory (T_M_) cells. Due to donor-reactive T cells, the rejection of the majority of second organ transplantations is more intense than the first transplantation (4–6). Human and animal studies have indicated that alloreactive T_M_ cells are an important part of the barrier, and understanding the underlying mechanisms may aid the inhibition of the rejection response and induce transplant tolerance.

To date, animal models of retransplantation have been studied less compared with animal models of transplantation, since the majority of the recipient mice fail to survive two surgeries, particularly in solid organ retransplantation with blood vessel anastomosis. In the present study, through the improvement of microscopic surgery skills, a consecutive skin-heart retransplant mouse model was established. The model was based on the rationale that the previous alloskin was a potent immunogen that vigorously induced alloantigen-specific T_M_ cells, which thereafter ‘at the second exposure’ promptly and vigorously launched an alloresponse upon the cardiac allograft following blood reperfusion (7). We hypothesized that this retransplantation mouse model may yield preclinical results that more closely predict clinical outcomes.

It is well documented that the activation and secretion of cytokines and chemokines regulate the recruitment of inflammatory cells and lead to the upregulation in the expression of cell-adhesion molecules (8). Previous studies on experimental and clinical transplants have demonstrated that chemokines play a critical role in the activation of innate immunity (9), ischemia-reperfusion injury (10) and the induction of adaptive immune responses (11). Increased expression levels of C-X-C motif chemokine ligand (CXCL) 9 and CXCL10 in a rat transplantation model have been shown to be accompanied by T-cell recruitment (12). In addition, alloreactive T_M_ cells have been previously demonstrated to contribute to increased expression and secretion of RANTES, as well as the migration of T_M_ and other inflammatory cells into the graft. With regard to previous observations, the present study investigated the effect of CXCL9 and CXCL10 in a mouse retransplantation model. As a result of previous allograft rejection, a retransplantation model is more complicated and exhibits a higher risk compared with primary transplantation.

## Materials and methods

### Mice

Female adult BALB/c and C57BL/6 mice (weight, 18–20 g; age, 8–10 weeks) were purchased from the Shanghai Laboratory Animal Center (Shanghai, China), and used as donors and recipients, respectively. All the mice were maintained under specific pathogen-free conditions and the experimental procedures were conducted in compliance with the Institutional Animal Care and Use guidelines. The study was approved by the ethics committee of the First Affiliated Hospital of Xiamen University, Xiamen, China.

### Groups

In the experimental group (n=6), cardiac transplantation was performed six weeks following skin grafting. In the control group (n=6), cardiac transplantation was performed without skin grafting. Untreated mice (n=6; C57BL/6) served as blank controls.

### Skin grafting

Full-thickness skin grafts were obtained from the lateral thoracic skin of the BALB/c mice and were cut into squares or rectangles of 1–1.5 cm^2^. Donor skin was engrafted onto the lumbar regions of the C57BL/6 mice and was assessed daily.

### Heterotopic cardiac transplantation

Cardiac transplantation was performed six weeks following skin grafting. Standard methods of murine heterotopic intraneck (anastomosis of the vessels of the neck using a nonsuture cuff technique) cardiac transplantation from BALB/c donors to C57BL/6 recipients were performed as described previously (13). Donor hearts were assessed by daily palpation following surgery. Cessation of a palpable heartbeat was defined as graft rejection.

### Histological examination

Cardiac grafts were harvested at day three following cardiac transplantation and preserved with formalin. Paraffin-embedded transventricular tissue sections (5 μm) were stained with hematoxylin and eosin. A rejection score was assigned by examining the extent of leukocytic infiltration and the anatomical destruction of the myocytes, according to the International Society for Heart & Lung Transplantation (ISHLT) criteria (14,15).

### Enzyme-linked immunosorbent assay (ELISA)

Serum levels of CXCL9, CXCL10, interferon (IFN)-γ, interleukin (IL)-2, IL-10 and tumor growth factor (TGF)-β in the recipient mice were determined using ELISA kits (R&D Systems, Minneapolis, MN, USA). A standard curve was generated using known quantities of the purified recombinant murine cytokines.

### Quantitative polymerase chain reaction (qPCR)

Reverse transcription (RT) was performed with 1 μg total RNA, that had been isolated from the grafts, using TRIzol reagent (Invitrogen Life Technologies, Gaithersburg, MD, USA). PCR analysis of the RT product (2 μl) was performed using a real-time PCR System (Applied Biosystems, Inc., Warrington, UK) with SYBR Green I fluorescence and β-actin as the reference. The primer sequences used for qPCR were as follows: β-actin forward, 5′-CAT CCG TAA AGA CCT CTA TGC CAA C-3′ and reverse, 5′-ATG GAG CCA CCG ATC CAC A-3′; IFN-γ forward, 5′-CGG CAC AGT CAT TGA AAG CCT A-3′ and reverse, 5′-GTT GCT GAT GGC CTG ATT GTC-3′; IL-2 forward, 5′-GGA GCA GCT GTT GAT GGA CCT AC-3′ and reverse, 5′-AAT CCA GAA CAT GCC GCA GAG-3′; IL-10 forward, 5′-GAC CAG CTG GAC AAC ATA CTG CTA A-3′ and reverse, 5′-GAT AAG GCT TGG CAA CCC AAG TAA-3′; TGF-β forward, 5′-TGA CGT CAC TGG AGT TGT ACG G-3′ and reverse, 5′-GGT TCA TGT CAT GGA TGG TGC-3′; CXCL9 forward, 5′-TGT GGA GTT CGA GGA ACC CT-3′ and reverse, 5′-TGC CTT GGC TGG TGC TG-3′; and CXCL10 forward, 5′-AGA ACG GTG CGC TGC AC-3′ and reverse, 5′-CCT ATG GCC CTG GGT CTC A-3′.

### Statistical analysis

All the results are presented as the mean ± standard deviation. The Kaplan-Meier method was used to plot the allograft survival curve, while the log-rank test was performed to determine differences in survival data. Data comparisons were analyzed with the Student’s unpaired t-test. SPSS 13.0 (SPSS Inc., Chicago, IL, USA) and GraphPad Prism 5 (San Diego, CA, USA) were used for statistical analysis. P<0.05 was considered to indicate a statistically significant difference.

## Results

### Survival times of the cardiac allografts

**Figure 1:**
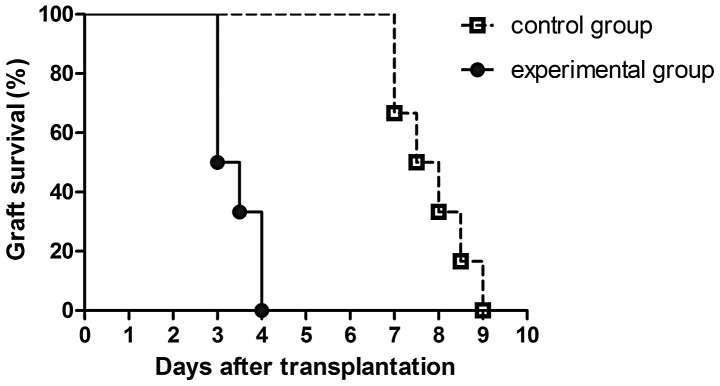
Donor hearts from BALB/c mice were transplanted into C57BL/6 mice, with or without skin grafting, and divided into experimental and control groups (n=6 per group). A log-rank test was performed to determine the differences in graft survival between the groups.

In the experimental group, the median survival time (MST) of the cardiac allografts was significantly shorter compared with the controls (P<0.001). As shown in Fig. 1, the MST was 7.75 days in the experimental group and 3.25 days in the control group.

### Histological evaluation

**Figure 2:**
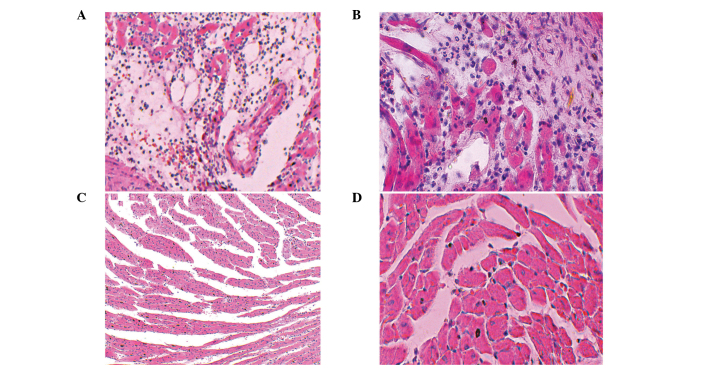
Histological evaluation of the cardiac allograft. Grafts were harvested on day three following cardiac transplantation and evaluated by H&E staining of the paraffin sections. (A and B) Experimental group showed acute rejection with extensive inflammatory cell infiltration, vascular injury and myocardial necrosis in the grafts (ISHLT grade 4; magnification, ×100 and ×400, respectively). (C and D) Grafts in the control group showed mild infiltration with no evident necrosis (ISHLT grade 1B-2; magnification, ×100 and ×400, respectively). H&E, hematoxylin and eosin; ISHLT, International Society for Heart & Lung Transplantation.

Compared with the control group, the destruction of cardiac allografts in the experimental group was more intense. The cardiac allografts were harvested from the transplanted mice on day three following transplantation for histological examination. As shown in Fig. 2, the experimental group was assigned an ISHLT grade of 4 (Fig. 2), while the control group was assigned an ISHLT grade of 1B-2 (Fig. 2), based on the extent of inflammatory cell infiltration and the destruction of the cardiac allografts in the two groups.

### CXCL9 and CXCL10 gene expression and key effector molecules in the cardiac grafts

**Figure 3:**
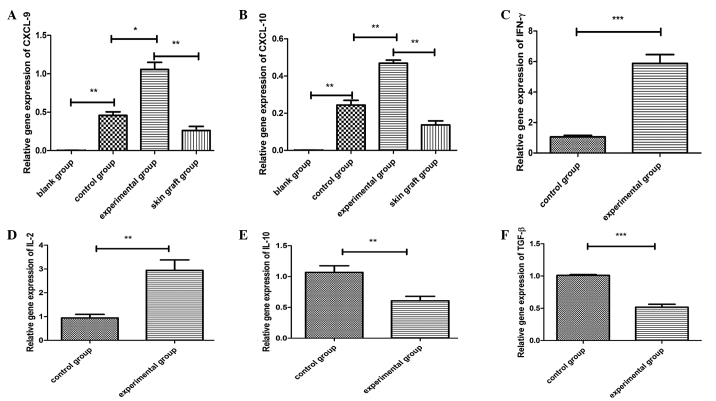
Relative gene expression levels of (A) CXCL9, (B) CXCL10, (C) IFN-γ, (D) IL-2, (E) IL-10 and (F) TGF-β in the cardiac allografts of the recipient mice. ^*^P<0.05; ^**^P<0.01; ^***^P<0.001; IFN, interferon; IL, interleukin; TGF, transforming growth factor; CXCL, C-X-C motif chemokine ligand.

RNA was isolated from the cardiac grafts on day three following the transplantation in recipient mice, and the cDNA products were synthesized and analyzed by qPCR. As shown in Fig. 3, statistically significant differences with regard to cytokine gene expression were observed among the groups (blank vs. control; control vs. experimental; skin graft vs. experimental). As shown in Fig. 3, the relative gene expression levels of IFN-γ and IL-2 were higher in the experimental group than in the control group, whereas the relative gene expression levels of IL-10 and TGF-β were lower in the experimental group compared with the control group.

### Effect of retransplantation on the secretion of CXCL9, CXCL10 and key effector molecules in the serum

**Figure 4:**
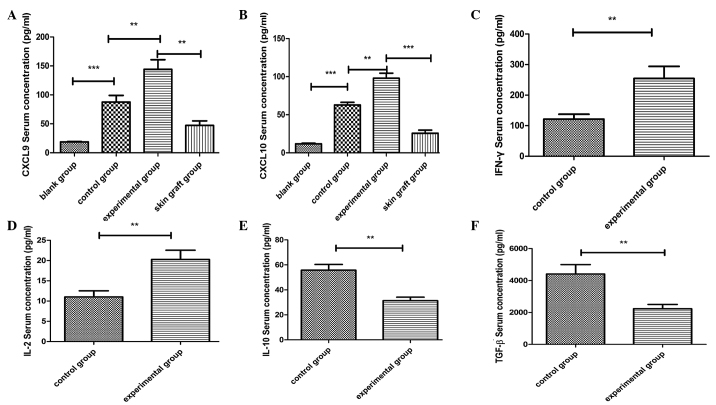
Serum concentrations of (A) CXCL9, (B) CXCL10, (C) IFN-γ, (D) IL-2, (E) IL-10 and (F) TGF-β in the recipient mice were determined by ELISA on day three following cardiac transplantation. ^**^P<0.01; ^***^P<0.001; CXCL, C-X-C motif chemokine ligand; ELISA, enzyme-linked immunosorbent assay; IFN, interferon; IL, interleukin; TGF, transforming growth factor.

ELISA was performed using sera obtained from the recipient mice at day three following cardiac transplantation. As shown in Fig. 4, statistically significant differences were observed in the cytokine expression levels between the groups (blank vs. control; control vs. experimental; skin graft vs. experimental). Furthermore, the expression levels of CXCL9 and CXCL10 in the serum were shown to correlate with the development of acute rejection. In addition, in the experimental group, the serum concentrations of IFN-γ and IL-2 were higher, while the concentrations of TGF-β and IL-10 were lower when compared with the control group. These observations indicated that the retransplantation model was more prone to the development of acute rejection compared with the transplantation model.

## Discussion

Acute rejection of allografts is a major complication following transplantation and is characterized by intragraft infiltration of activated mononuclear cells. However, the mechanism of leukocyte recruitment has not been fully established. In recent years, an increasing number of studies have demonstrated that the activation and secretion of chemokines regulate inflammatory cell recruitment and lead to the upregulation of cell-adhesion molecule expression (8–11,16), thus, revealing the critical role of chemokines in the development of acute rejection (8). Chemokines are a group of low-molecular weight (8–11 kDa) cytokines that mediate cellular trafficking and can be divided into four families (C, CC, CXC and CX3C) based on conserved cysteine residues in the amino-terminal end of the molecule. CXCL9/MIG (a monokine induced by IFN-γ) and CXCL10/IP-10 (IFN-γ-inducible protein 10) belong to the CXC chemokine subgroup of the chemokine superfamily. CXCL9 and CXCL10 bind to their shared receptor, CXCR3, which is expressed on Th1 cells, a number of B cells and natural killer cells, and participate in lymphocyte recruitment in virtually all stages following transplantation. In human and animal studies, CXCL9 and CXCL10 expression levels have been shown to be elevated in solid organ rejection, including skin, heart, renal and lung (16–19). In addition, neutralization of CXCL9 and CXCL10 has been shown to prolong the allograft survival time by reducing the infiltration of mononuclear cells and attenuating the acute rejection. However, the role of CXCL9 and CXCL10 in retransplantation models remains unknown. The model used in the present study is known to closely mimic the clinical setting, as a quicker and more vigorous alloresponse is induced compared with other models (20). In the present study, this retransplantation model was used to determine whether the levels of CXCL9 and/or CXCL10 increase post-transplantation. Previous research using this model indicated that it is an effective method to acquire T_M_ cells in small mice following skin grafting (21). In contrast to naïve T cells, T_M_ cells are programmed to activate quickly, with a reduced requirement for costimulatory signals and a lower threshold for activation (1). Alloreactive T_M_ cells are insensitive to conventional immunosuppressive treatments, including rapamycin or tacrolimus, indicating that T_M_ cells may induce a faster and stronger immune response in terms of proliferation and IFN-γ secretion on reexposure to the same antigen. As shown in Figs. 3 and 4, gene expression and serum concentration levels of CXCL9 and CXCL10 in the experimental group were markedly higher compared with the control group. Similarly, the infiltration of mononuclear cells was more intense in the experimental group than in the control group following cardiac allograft transplantation (Fig. 2). Injury to the graft was predominantly due to the graft-infiltrating T cells that produced IFN-γ, affecting the function of the donor graft. CXCL9 and CXCL10 can traffic Th1 cells, and other inflammatory cells migrate throughout the recipient’s blood stream to the graft and are activated to express effector functions. Prior to heterotopic cardiac transplantation, the serum concentration and gene expression levels of CXCL9 and CXCL10 were higher in the transplantation group compared with the blank control group; similarly, levels of other cytokines, including IFN-γ and IL-2, were higher in the transplantation group compared with the blank control group. Furthermore, the MST of the cardiac allografts was significantly shorter in the experimental group (3.25 days) compared with the control group (7.75 days). Therefore, CXCL9 and CXCL10 may serve as possible biomarkers to predict the development of acute rejection following transplantation. The observations of the present study are supported by the evidence that chemokines play a pivotal role in directing immune cell differentiation, migration and proliferation during acute rejection; therefore, their circulating levels in the serum and plasma are strictly associated with the immune status of the patient (22,23). Compared with biopsy samples, circulating cytokine concentrations can be easily assessed from serum collected from the peripheral blood of a transplant patient. However, further studies are required to investigate additional factors that influence the circulating levels of chemokines *in vivo* and/or *in vitro*.

As stated previously, T_M_ cells are not sensitive to conventional immunosuppressive regimens (24). A number of studies have demonstrated that the use of anti-CXCL9 and anti-CXCL10 antibodies can prolong allograft survival time (25,26). Thus, further research is required to determine whether anti-CXCL9 and/or anti-CXCL10 molecules may be an effective approach to prevent T_M_ cells and other inflammatory cells from infiltrating into the grafts, alleviating acute retransplant rejection.

Collectively, the results of the present study indicate that compared with naïve T cells, T_M_ cells induce higher levels of CXCL9 and CXCL10, which in turn leads to increased chemokine-mediated inflammatory cell trafficking into the grafts, aggravating the development of acute rejection. The differences in the levels of CXCL9 and CXCL10 between pre- and post-transplant mice indicate that CXCL9 and CXCL10 may be used as possible biomarkers to predict acute rejection. Future studies should determine whether strategies of inhibiting CXCL9 and CXCL10, combined with other immunosuppressive treatments, can alleviate acute retransplant rejection.
